# Predictors Associated with Forgotten Knee in Patients with Total Knee Arthroplasty Based on Multivariable Linear Regression

**DOI:** 10.1111/os.13959

**Published:** 2023-12-04

**Authors:** Peifang Li, Jingying Xie, Ning Ning, Jiali Chen

**Affiliations:** ^1^ Department of Orthopedic Surgery, West China Hospital Sichuan University/West China School of Nursing, Sichuan University Chengdu China

**Keywords:** Forgotten Joint Score, Multivariable Linear Regression, Total Knee Arthroplasty

## Abstract

**Objective:**

Existing studies have conflicting results about the predictors of forgotten joints in patients with total knee arthroplasty (TKA), and the relationship between psychosocial factors and forgotten knees is unknown. The purpose of this study was to confirm predictors for the forgotten joint in TKA patients.

**Methods:**

This was an observational, prospective longitudinal study. A total of 205 patients who underwent TKA and a 6‐month follow‐up were included between August 2020 and September 2021. Demographic characteristics, clinical characteristics, and psychosocial variables were collected before TKA surgery (T0). The forgotten joint score (FJS) was taken before TKA surgery (T0) and at 1 month (T1), 3 months (T3), and 6 months (T6) after TKA surgery. The psychosocial variables were also completed at T6. Bivariate and multivariable linear regressions (LR) were performed to screen the predictors associated with FJS (T6).

**Results:**

Patients who underwent TKA in our study had a mean FJS of 20.3 ± 12.2 before surgery, 15.9 ± 10.3 at 1 month, 28.7 ± 12.6 at 3 months, and 40.3 ± 12.5 at 6 months. The predictors were sex, combined musculoskeletal disorders (MSD), operation time, FJS (T3), range of motion (ROM) (T6), pain score (T6), Groningen orthopaedic social support scale (GO‐SSS) score (T6), and the generalized anxiety disorder scale (GAD) score (T6). The data satisfied the assumptions of multivariable linear regressions. The multiple *R* ^2^of LR was 0.71, and the adjusted *R* ^2^ was 0.70. The *F*‐statistic of the LR model was 59.5 (*p* < 0.000).

**Conclusion:**

Our study revealed the level of forgotten knee decreased slightly from preoperation to 1 month postoperatively and then increased from 1 month postoperatively to 6 months postoperatively in TKA patients. The main predictors associated with the FJS at 6 months after surgery were sex, combined MSD, operation time, FJS (T3), ROM (T6), pain score (T6), GO‐SSS score (T6), and anxiety (T6).

## Graphical abstract

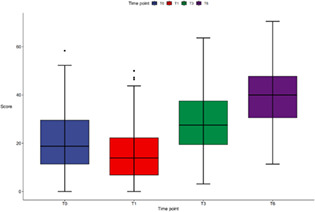

## Introduction

Knee osteoarthritis (KOA) is a degenerative bone and joint disease in the elderly population. It has a heavy disease burden and a high rate of late disability and seriously affects daily living.^1^, ^2^ Total knee arthroplasty (TKA) is an effective treatment for end‐stage KOA.^3^ The ultimate goal of joint replacement is to restore the function of the joint so that the patient “forgets” their artificial joint because it feels like a natural joint.^4^ Therefore, in addition to the physician's assessment outcomes, patient‐reported outcome measurements (PROMs) are crucial in assessing the patient's experience and perception after surgery.

The forgotten joint score (FJS), as an important PROM instrument to measure the patient's ability to forget artificial joints in everyday life, was developed and validated by Behrend *et al*.^5^ It has been demonstrated that the FJS shows better responsiveness and less of a ceiling effect than previous PROMs of TKA patients, which suggests an advantage of FJS in assessing patients with good outcome.^4^, ^6^ Patients with score >89 on the FJS‐12 were considered to have forgotten the joint operated on during TKA.^7^ Peersman *et al*. found that patients who underwent TKA had a mean FJS of 0.9 (95% CI, −5.4‐7.02) at 2 weeks, 19.1 (95% CI, 14.9–23.3) at 12 weeks, and 39.6 (95% CI, 34.7–44.4) at 6 months.^8^

Understanding the predictors of forgotten joints is a prerequisite for guiding decision‐making to improve the patient's ability to forget the operated joint after surgery and enhance recovery. Nielsen *et al*.^9^ showed that the level of forgotten joint in patients who received primary TKA was affected by age, sex, preoperative knee alignment, and osteoarthritis severity. Behrend identified three predictors of forgotten knee after TKA: age, body mass index (BMI), and sex.^10^ However, Eymard *et al*.^11^ found in a 6‐year follow‐up of 423 patients who underwent TKA that sex, age, BMI, preoperative pain, functional limitations, and patellar resurfacing were not significantly correlated with forgotten knee, while depression at baseline, presence of patellar subluxation after surgery, active flexion, preoperative patellar pain, and postoperative patellar subluxation were associated with forgotten knee.

At present, existing studies have conflicting results about the physiological predictors of forgotten joints in patients who have undergone TKA, and the relationship between psychosocial factors and forgotten knees is unknown. The predictors of forgotten joints are unknown in Chinese TKA patients.

Therefore, the aim of this prospective study were as follows: (i) to investigate the forgotten joint status of TKA patients before surgery 1 month after surgery, 3 months after surgery, and 6 months after surgery; (ii) to construct a linear regression (LR) to identify the predictors for the level of forgotten joint in TKA patients at 6 months after surgery.

## Materials and Methods

All the patients provided informed consent and were advised that they were free to withdraw from the study at any time. All data were anonymous or kept confidential. Our study was approved by the Ethics Committee on Biomedical Research, West China Hospital of Sichuan University (IRB/IEC number: 2019809) and registered at https://www.chictr.org.cn/.

### Design and Population

This was an observational, prospective longitudinal study. The study was carried out in the department of orthopaedics of a tertiary care hospital in Sichuan Province between August 2020 and September 2021. All the patients who were diagnosed with KOA, underwent TKA by the same surgeon and agreed to participate in this study were included. The study inclusion criteria were (i) aged older than 18 years, (ii) met the diagnostic criteria for osteoarthritis of the knee as described in the Chinese guidelines for the diagnosis and treatment of osteoarthritis (2021 edition), (iii) underwent unilateral TKA under general anesthesia during hospitalization (initial TKA on the operated side), and (iv) had the ability to understand verbal and written information in Chinese. Exclusion criteria were (i) rheumatoid arthritis, gouty arthritis, hemophilic arthritis, traumatic arthritis, joint tuberculosis, and ankylosing spondylitis (ii) severe valgus or valgus deformity (>20°) of the knee on the operated side, and (iii) malignancy or severe organ dysfunction.

### Study Variables

We collected the patients’ demographic characteristics, clinical characteristics, and psychosocial variables. The demographic characteristics included sex, age, marriage, BMI, education, and occupation. Clinical characteristics incorporated primary TKA or not, American Society of Anesthesiology (ASA) score, intraoperative blood loss, operation time, intraoperative use of tourniquet, comorbidities including musculoskeletal disorders (MSD), hypertension, diabetes, flexion deformity, internal and external inversion, range of motion (ROM), and pain before surgery and at 6 months after surgery, which were noted by the investigators after consulting the medical records. Other psychosocial variables included perceived social support, expectations about surgery, and anxiety and depression before surgery and at 6 months after surgery, assessed by the Groningen Orthopaedic Social Support Scale (GO‐SSS),^12^ the Hospital for Special Knee Replacement Expectations Surveys (HSS‐KRES),^13^ the Generalized Anxiety Disorder scale (GAD‐7),^14^ and the Patient Health Questionnaire (PHQ‐9),^15^ respectively.

### Forgotten Joint Score

The FJS is a valid patient‐reported outcome measure used to assess joint awareness in the hips and knees during various activities of daily living.^5^ FJS consists of 12 questions and is scored using a five‐point Likert response format with the raw scores transformed to range from 0 to 100 points. Higher scores indicate a more favorable outcome; that is, a more natural artificial joint and a high likelihood of forgetting the affected joint in daily life. In its validation study, it showed a low ceiling effect and high internal consistency (Cronbach's alpha was 0.95) and discriminated well between good, very good, and excellent outcomes after joint arthroplasty.^16^ The scale has now been validated in multiple languages. The Chinese version of the FJS was introduced by Cao *et al* ^17^ It has good internal consistency (Cronbach's alpha was 0.907) and test–retest reliability (ICC was 0.970). In our study, Cronbach's alpha was 0.711.

### Data Collection

Before data collection, the primary investigator provided unified training for the team, including the overall study content, questionnaire composition, patient inclusion and exclusion criteria, questionnaire collection process, and communication precautions, to ensure the reliability of the data. The purpose and significance of this study were explained to each patient and their family before administering the questionnaire survey, and the patients who fully understood the matters and inclusion criteria of the study were included. During hospitalization, data were collected from the orthopaedic inpatient ward. Follow‐up data were collected through uniform telephone follow‐up (to ensure the collection of subsequent follow‐up data, at least two telephone numbers were reserved to reach the patients, and the patients were informed of the purpose of providing two telephone numbers for the follow‐up). We collected the patients’ demographic characteristics, clinical characteristics, and psychosocial variables before TKA surgery (T0). The FJS was obtained before TKA surgery (T0) and at 1 month (T1), 3 months (T3), and 6 months (T6) after TKA surgery. The GO‐SSS, HSS, GAD‐7, and PHQ‐9 were completed at T6.

### Statistical Analysis

A descriptive statistical analysis of each variable was performed. The Kolmogorov–Smirnov test was used to check the normal distribution of data. Absolute and relative frequencies were calculated for the qualitative variables, mean and standard deviation (SD) for the normal distribution quantitative variables, and quartile for the abnormal distribution quantitative variables. Analysis of variance was used to analyze the differences of TKA patients at different times and Bonferroni tests were used for multiple‐comparison between any two time points. To determine the relationship between the different factors and FJS (T6), the B, 95% confidence interval (CI95%), and *p*‐value were calculated by linear regression. Furthermore, a multivariable linear regression (MLR) analysis was carried out to construct a predictive model. The automatic backward and forward stepwise procedure was used for the multivariable analysis. Residual diagnostic plots, variance inflation factor (VIF) tests, and comprehensive verification were performed to diagnose whether the model satisfied the assumptions of MLR. The *R* ^2^, adjusted *R* ^2^, *F*‐statistic and *p*‐value of the model were calculated to evaluate its predictive capacity.

The above statistical methods were carried out with R software version 4.1.3 (Lucent Technologies USA) with the R software packages “car,” “multtest,” “dplyr,” “epiDisplay,” “stats,” “gvlam,” “rinds,” and “ggplot2”. A *p*‐value < 0.05 was considered significant.

## Results

### Sample Characteristics

Initially, 227 patients who met the inclusion and exclusion criteria were investigated. Of these, one patient was unable to be contacted. Eleven patients who underwent contralateral TKA within 6 months after primary surgery and 10 patients with medical comorbidities that would have confounded the results of this study were excluded. In total, 205 patients (90.31%) completed all follow‐ups. Among the included patients, 82% were women, the median age was 68 years old, and the median body mass index (BMI) was 25.04 kg/m^2^. The other available demographics are presented in Table 1.

**TABLE 1:** Sociodemographic and clinical characteristics of the study sample

Variable	Mean (SD)/*n* (%)/quartile	Bivariate	*p*	Multivariate	*p*
		B (95% CI)		B (95% CI)	
Gender					
Male	37 (18)	Reference		Reference	
Female	168 (82)	−12.74 (−16.87, −8.60)	0.00	−5.20 (−7.85, −2.55)	0.000
Age					
P_50_ (P_25_, P_75_)	68 (63, 72)	0.003 (−0.24, 2.41)	0.98		
Marriage					
Married	163 (79.5)	Reference			
Divorced	5 (2.4)	−4.31 (−15.56, 6.93)	0.45		
Widowed	37 (18)	1.05 (−3.46, 5.56)	0.65		
BMI					
P_50_ (P_25_, P_75_)	25.04 (22.99, 27.25)	0.08 (−0.03, 0.19)	0.147		
Education					
Illiteracy	42 (20.5)	Reference			
Primary school	69 (33.7)	0.42 (−4.37, 5.20)	0.86		
Middle school	47 (22.9)	4.37 (−0.82, 9.57)	0.10		
College or above	29 (14.1)	5.34 (0.15, 10.53)	0.04		
Occupation					
No	24 (11.7)	Reference			
Manual workers	122 (59.5)	2.40 (−3.09, 7.88)	0.39		
Mental worker	59 (28.8)	5.65 (−0.30, 11.60)	0.06		
Year of OA					
P_50_ (P_25_, P_75_)	10 (5, 14.5)	0.03 (−0.22, 0.27)	0.835		
Concomitant chronic disease					
No	11 (5.4)	Reference			
Yes	194 (94.6)	−9.32 (−16.89, −1.76)	0.02		
MSD					
No	52 (25.4)	Reference		Reference	
Yes	153 (74.6)	−5.61 (−9.51, −1.72)	0.005	−1.82 (−4.04, 0.41)	0.11
Osteoporosis					
No	106 (51.7)	Reference			
Yes	99 (48.3)	−3.53 (−6.96, −0.10)	0.04		
Hypertension					
No	105 (51.2)	Reference			
Yes	100 (48.8)	0.06 (−3.40, 3.52)	0.002		
Diabetes					
No	172 (83.9)	Reference			
Yes	33 (16.1)	−1.26 (−5.96, 3.44)	0.59		
Primary TKA					
No	51 (24.9)	Reference			
Yes	154 (75.1)	−1.87 (−5.86, 2.13)	0.36		
Flexion deformity					
No	69 (33.7)	Reference			
Yes	136 (66.3)	−1.74 (−4.40, 2.92)	0.69		
Internal and external inversion					
No	64 (31.2)	Reference			
Yes	141 (68.8)	0.48 (−3.25, 4.21)	0.8		
ASA score					
1	1 (0.5)	Reference			
2	155 (75.6)	−11.43 (−36.09, 13.24)	0.36		
3	49 (23.9)	−14.94 (−39.78, 9.90)	0.24		
Intraoperative blood loss					
P_50_ (P_25_, P_75_)	100 (90, 100)	0.02 (−0.03, 0.07)	0.523		
Operation time					
P_50_ (P_25_, P_75_)	77 (67, 90)	0.13 (0.03, 0.22)	0.01	0.05 (−0.01, 0.10)	0.10
Intraoperative use of tourniquet					
P_50_ (P_25_, P_75_)		−0.36 (−3.78, 3.06)	0.836		
ROM					
P_50_ (P_25_, P_75_)	110 (100, 110)	0.18 (−0.09, 0.46)	0.183		
Pain					
0	8 (3.9)	Reference			
1	138 (67.3)	−5.02 (−13.93, 3.89)	0.27		
2	58 (28.3)	−8.98 (−18.22, 0.26)	0.06		
3	1 (0.5)	−13.78 (−39.76, 12.21)	0.30		
FJS (T3)					
Mean (SD)	28.7 (12.6)	0.74 (0.65, 0.83)	0.000	0.48 (0.38, 0.57)	0.000
ROM (T6)					
P_50_ (P_25_, P_75_)	110 (100, 120)	0.41 (0.29, 0.54)	0.000	0.11 (0.15, 0.61)	0.01
GO‐SSS (T6)					
P_50_ (P_25_, P_75_)	44 (42, 46)	1.14 (0.79, 1.50)	0.000	0.38 (0.15, 0.61)	0.001
Pain (T6)					
0	165 (80.5)	Reference		Reference	
1	40 (19.5)	−17.06 (−20.73, −13.39)	0.000	−8.22 (−11.02, −5.43)	0.000
GAD (T6)					
No	178 (86.8)	Reference			
Yes	27 (13.2)	−14.02 (−18.75, −9.28)	0.000	−2.30 (−5.48, 0.89)	0.16
PHQ (T6)					
No	151 (73.7)	Reference			
Yes	54 (26.3)	−14.26 (−17.69, −10.87)	0.000		

Factors associated with FJS at 6 month in TKA patients. Bivariate and multivariate analysis by linear regression.

Abbreviations: ASA, American Society of Anesthesiology score; BMI, body mass index; FJS, forgotten joint score; GAD, generalized anxiety disorder score; GO‐SSS, Groningen orthopaedic social support scale; MSD, musculoskeletal disorders; OA, osteoarthritis; PHQ, patient health questionnaire; ROM, range of motion; TKA, total knee arthroplasty.

### Forgotten Joint Score at Different Time Points

**FIGURE 1:**
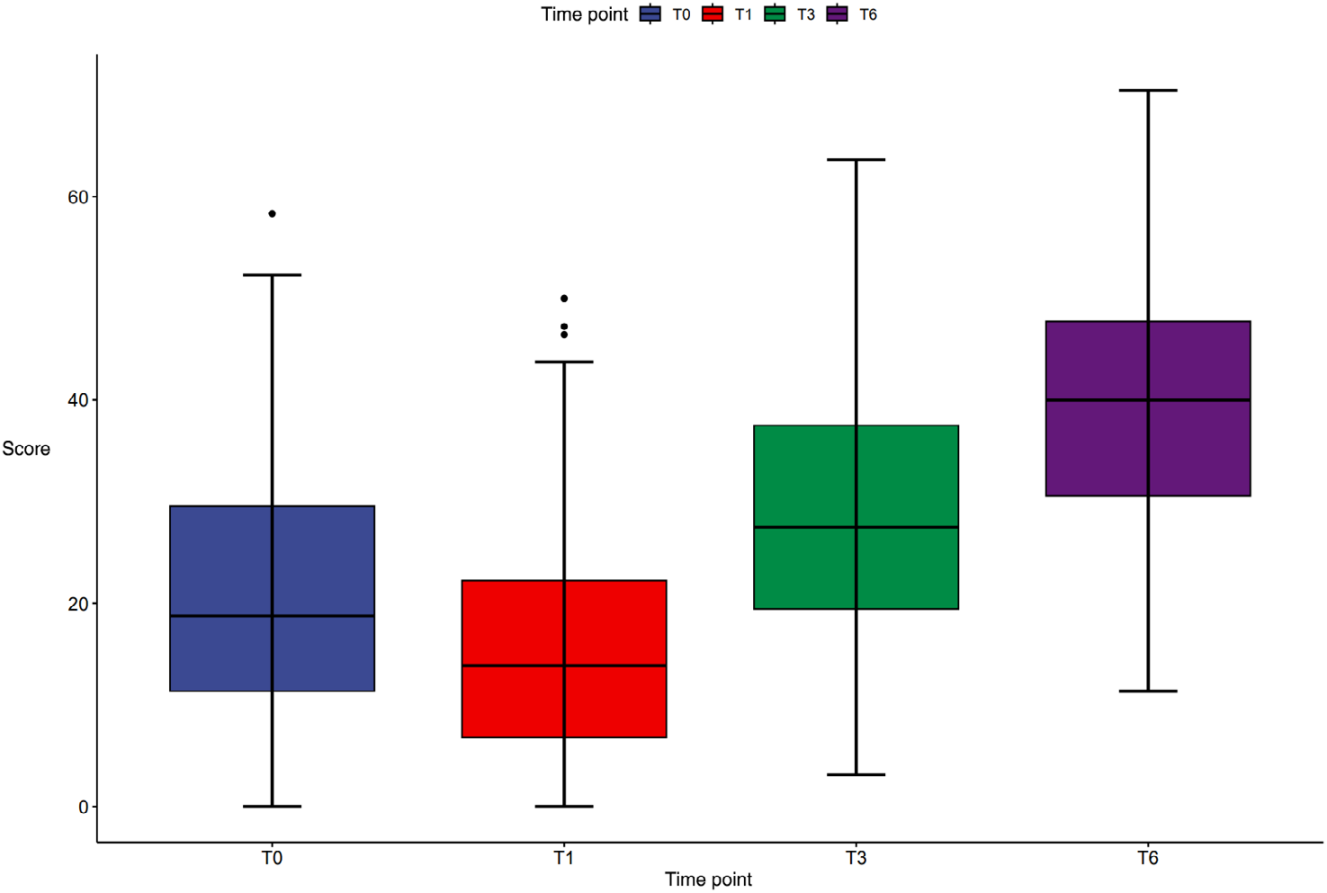
FJS scores at baseline (T0), 1 month (T1), 3 months (T3), and 6 months (T6).

The patients who underwent TKA in our study had a mean FJS of 20.3 ± 12.2) before surgery, 15.9 ± 10.3 at 1 month, 28.7 ± 12.6 at 3 months, and 40.3 ± 12.5 at 6 months (Figure 1). The mean FJS showed that the level of forgotten knee decreased slightly from pre‐operation to 1 month postoperatively and then increased from 1 month postoperatively to 6 months postoperatively, and the increase was greater at 1 to 3 months postoperatively than at 3 to 6 months postoperatively.

The variance test showed that the FJS of the TKA patients at different periods were significantly different (*F* = 305.299, *p* < 0.001). Bonferroni tests were performed. The results showed that there were statistically significant differences in the FJS before the operation and at 1, 3 and 6 months after surgery (*p* < 0.001). Compared with the preoperative FJS, the FJS at 1 month after surgery decreased by 4.37 points (95% CI: 1.887–6.862), and the difference was statistically significant (*p* < 0.001). The FJS at 3 months after surgery increased by 11.56 points (95% CI: −15.084 to −10.613) when compared with 1 month after surgery, and the difference was statistically significant (*p* < 0.001). The FJS at 6 months after surgery increased by 12.85 points (95% CI: −13.239 to −9.884) when compared with 1 month after surgery, and the difference was statistically significant (*p* < 0.001).

### Bivariate and Multivariable Analyses by Linear Regression

In the bivariate analysis, the FJS (T6) was the dependent variable and the demographic characteristics, clinical characteristics, FJS (T3), and psychosocial factors (T6) were the independent variables. The results showed that sex, education, concomitant chronic disease, combined MSD, combined osteoporosis, combined hypertension, operation time, pain (T0), FJS (T3), ROM (T6), pain score (T6), GO‐SSS score (T6), GAD‐7 score (T6), and PHQ‐9 score (T6) were associated with statistically significant differences in the FJS (T6) (Table 1). We performed a multivariable linear regression (MLR) analysis of all the factors that had a *p*‐value <0.05 in the bivariate analysis. The predictive model was constructed with eight predictors. The predictive factors were sex, combined MSD, operation time, FJS (T3), ROM (T6), pain score (T6), GO‐SSS score (T6), and GAD‐7 score (T6) (Table 1). The multiple *R* ^2^ of this model was 0.71, and the adjusted *R* ^2^ was 0.70. The *F*‐statistic of this model was 59.5 (*p* < 0.000).

### The Diagnosis of the Model

**FIGURE 2:**
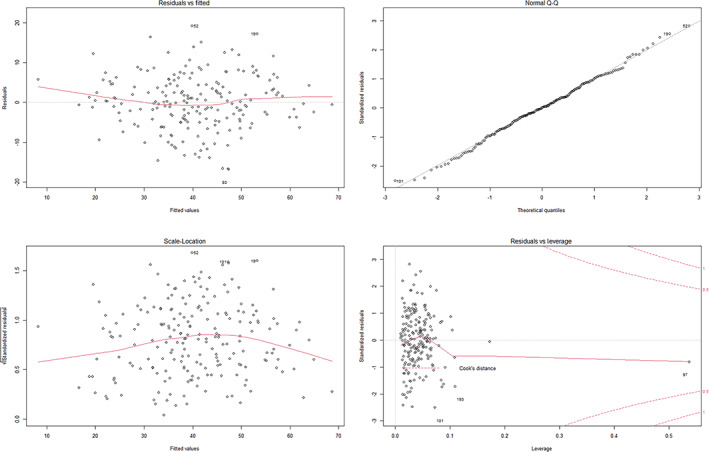
Diagnostic plots for FJS (T6) following multiple linear regression showing residuals versus fitted values, normal Q–Q, scale location and residuals versus leverage plots.

Inspection of the diagnostic plots showed that the residuals of the model were independent of the fitted values and randomly distributed (Figure 2A). The residuals were normally distributed, and the residuals were closely distributed along the straight line in the Q–Q plot (Figure 2B). The Shapiro–Wilk normality test also showed that the residuals were normally distributed (W = 0.99, *p* value = 0.79). Furthermore, the scale‐location plots suggested that the residuals were homogeneous in variance (Figure 2C). Residuals versus leverage plots did not indicate any serious abnormity in outlier, high leverage, and influent plots (Figure 2D). The VIFs of the predictive factors screened by MLR were less than 4 (Table 2) and showed that the independent variables were not multicollinear. The hypothesis of the linear model was verified comprehensively, and the results showed that the global stat, skewness, kurtosis, link function, and heteroscedasticity were acceptable (*p* > 0.05) (Table 3). Therefore, the model satisfied the assumptions of MLR. It was concluded that the predictive model was valid.

**TABLE 2:** The VIF of the predictive factors

Value	Gender	MSD	Operation time	FJS (T3)	ROM (T6)	GO‐SSS (T6)	Pain (T6)	GAD (T6)
VIF	1.15	1.04	1.08	1.48	1.21	1.17	1.36	1.28

Abbreviations: FJS, forgotten joint score; GAD, generalized anxiety disorder score; GO‐SSS, Groningen orthopaedic social support scale; MSD, musculoskeletal disorders; ROM, range of motion; VIF, variance inflation factor.

**TABLE 3:** Comprehensive verify of the model

	Value	*p*‐value	Decision
Global stat	6.05	0.20	Assumptions acceptable
Skewness	0.01	0.92	Assumptions acceptable
Kurtosis	0.02	0.90	Assumptions acceptable
Link function	4.22	0.04	Assumptions acceptable
Heteroscedasticity	1.80	0.18	Assumptions acceptable

## Discussion

In our study, we found the level of forgotten knee decreased slightly from pre‐operation to 1 month postoperatively and then increased from 1 month postoperatively to 6 months postoperatively in TKA patients. The main predictors associated with the FJS at 6 months after surgery were sex, combined MSD, operation time, FJS (T3), ROM (T6), pain score (T6), the patients’ perceived social support (T6), and anxiety (T6).

### Comparison of Forgotten Knee Level with Patients after Total Knee Arthroplasty

Our study showed that the mean FJS scores in the patients who underwent TKA were 28.7 ± 12.6 at 3 months and 40.3 ± 12.5 at 6 months. Peersman *et al*. (2019) indicated that patients who underwent TKA had a mean FJS of 19.1 (95% CI, 14.9–23.3) at 12 weeks and 39.6 (34.7–44.4) at 6 months.^7^ Singh *et al*. (2021) found that the mean FJS scores in the patients who underwent TKA were 24.66 at 3 months after surgery and 40.95 at 1 year after surgery.^18^ The FJS scores of TKA patients at 3 months in our study was the highest because the TKA techniques were advanced and enhanced recovery after surgery (ERAS) measures were implemented during the perioperative period, such as reducing intraoperative bleeding, multimodal analgesia, preventing deep vein thrombosis (DVT), and early postoperative functional exercise. The level of the TKA patients who forgot the knee at 6 months after TKA was similar, implying that the patients recovered quickly within 3–6 months after surgery. Therefore, we should follow up the patient's recovery within 6 months after surgery.

### Predictors Associated with Forgotten Knee in Total Knee Arthroplasty Patients

We observed that female sex had a statistically significant relationship with a low FJS, which was similar to that found in the study by Behrend, while BMI and age were not associated with FJS. Behrend *et al*. (2016) concluded that younger age, female sex, and higher BMI had a statistically significant association with having more awareness of the operated knee at 1 year after surgery.^10^ However, the study by Eymard *et al*. (2017) found in a 6‐year follow‐up of 423 patients who underwent TKA that sex, age, and BMI were not significantly correlated with forgotten knee.^11^ Prior reports investigating the impact of demographic factors on TKA outcomes had contradictory findings. Possible explanations for this might be that the time at which the dependent variable was assessed was different in different studies.

Patients who had MSD complications and poor ROM at 6 months after surgery had a low FJS in our study. Eymard *et al*. (2017) also found that better active flexion predicted a higher FJS score.^11^ The possible reason is that a substantial limitation of range of motion leads to some limitation in performing daily living activities, thus increasing the patient's awareness of the joint.

We assessed anxiety and depression at 6 months after surgery and found that anxiety had a very negative influence. Previous studies have also shown a central role of depression in TKA outcomes.^19^, ^20^ Eymard *et al*. (2017) demonstrated that baseline depression was correlated with forgotten knee and might suppose a negative association with forgotten knee because they did not assess depression during the follow‐up.^11^ Patients with anxiety or depression after TKA pay too much attention to their knee, so the level of forgotten knee is low.

Pain at 6 months is also an important predictor that influenced a lower FJS. The pain made the patient aware of the presence of the artificial joint and reduced the level of the patient forgetting the joint. Determining the predictors of a patient's ability to “forget” the artificial joint can help formulate better preventive or therapeutic interventions. A low FJS in pain patients indicates that pain management are needed in patients with TKA.

### Strengths and Limitations

Our study was a prospective longitudinal survey of TKA patients, and few patients were loss to follow‐up. All TKAs were performed by the same surgeon, thus avoiding the risk of heterogeneity induced by different levels of experience, techniques, and perioperative management procedures. The results confirm the hypotheses of previous studies on the relationship between anxiety and FJS and prove the relationship between current psychosocial factors and FJS. The limitation of the present study was the 6‐month follow‐up. The time is short compared to many previous studies. We will further evaluate the patient's level of forgotten knee for a longer time in the future.

## Conclusions

In this study, the authors reported the level of forgotten knees before surgery and 1 month, 3 months, and 6 months after surgery, developed a predictive model, and provided important information concerning the demographic characteristics, clinical characteristics, and psychosocial factors that predicted the level of forgotten knees at 6 months after TKA. The main factors associated with the FJS at 6 months after surgery were sex, combined MSD, operation time, FJS (T3), ROM (T6), pain score (T6), GO‐SSS score (T6), and anxiety (T6).

## Ethics Statement

This clinical study was approved by the Ethics Committee on Biomedical Research, West China Hospital of Sichuan University.

## Author Contributions

PFL, JYX, and NN designed the study. PFL and JYX drafted the manuscript, collected the data, and conducted the data analysis. JLC and NN critically revised the manuscript for important intellectual content. All authors approved the final version of the manuscript.
